# Hot Deformation Behavior and Microstructure Evolution Mechanisms of Ti6Al4V Alloy under Hot Stamping Conditions

**DOI:** 10.3390/ma17112531

**Published:** 2024-05-24

**Authors:** Mingjia Qu, Zhengwei Gu, Xin Li, Lingling Yi, Yi Li, Ge Yu, Yafu Zhao

**Affiliations:** 1 State Key Laboratory of Automobile Materials, Jilin University, Changchun 130025, China; qumj21@mails.jlu.edu.cn (M.Q.); guzw@jlu.edu.cn (Z.G.); yill20@mails.jlu.edu.cn (L.Y.); henrylee@jlu.edu.cn (Y.L.); yuge@jlu.edu.cn (G.Y.); 2 Department of Materials Science and Engineering, Jilin University, Changchun 130025, China; 3 CRRC Changchun Railway Vehicles Co., Ltd., Changchun 130022, China

**Keywords:** titanium alloy, processing map, hot stamping, microstructure evolution, dynamic recrystallization

## Abstract

Through the study of the thermal rheological behavior of Ti6Al4V alloy at different temperatures (500 °C, 600 °C, 700 °C, and 800 °C) and different strain rates (0.1 s^−1^, 0.05 s^−1^, 0.01 s^−1^, and 0.005 s^−1^), a constitutive model was developed for Ti6Al4V alloy across a wide temperature range in the hot stamping process. The model’s correlation coefficient reached 0.9847, indicating its high predictive accuracy. Hot processing maps suitable for the hot stamping process of Ti6Al4V alloy were developed, demonstrating the significant impact of the strain rate on the hot formability of Ti6Al4V alloy. At higher strain rates (>0.05 s^−1^), the hot processing of Ti6Al4V alloy is less prone to instability. Combining hot processing maps with hot stamping experiments, it was found that the forming quality and thickness uniformity of parts improved significantly with the increase in stamping speed. The phase composition and microstructures of the forming parts under different heating temperature conditions have been investigated using SEM, EBSD, XRD, and TEM, and the maximum heating temperature of hot stamping forming was determined to be 875 °C. The recrystallization mechanism in hot stamping of Ti6Al4V alloys was proposed based on EBSD tests on different sections of a hot stamping formed box-shaped component. With increasing deformation, the effect of dynamic recrystallization (DRX) was enhanced. When the thinning rate reached 15%, DRX surpassed dynamic recovery (DRV) as the dominant softening mechanism. DRX grains at different thinning rates were formed through both discontinuous dynamic recrystallization (DDRX) and continuous dynamic recrystallization (CDRX), with CDRX always being the dominant mechanism.

## 1. Introduction

In recent years, titanium alloys have been widely used in the aerospace and transportation industries, attributed to their high specific strength and corrosion resistance [1,2]. The application of titanium alloy products in major transportation vehicles like airplanes and trains reduces both self-weight and the frequency of component replacement [3,4], driving an increasing demand for titanium alloy machining processes characterized by low manufacturing costs, short machining cycles, and high forming quality. However, the poor plasticity and significant springback of titanium alloy at room temperature make its forming challenging [5]. Consequently, plastic processing of titanium alloys is typically performed at high temperatures. Conventional hot working processes for titanium alloy sheets involve superplastic forming [6] and isothermal hot press forming. However, superplastic forming which requires blow molding of the sheet at high temperatures, imposes stringent requirements on forming equipment. Although isothermal hot press forming uses relatively simple forming equipment, it necessitates heating the forming tools, leading to short die life and considerable energy consumption. Additionally, both superplastic forming and isothermal hot press forming are relatively time consuming, with dies requiring several hours of preheating before use, which diminishes production efficiency. These factors are among the reasons that hinder the widespread application of titanium alloy components. Therefore, identifying environmentally friendly, efficient, and high-quality processing methods has become a current research focus.

The hot stamping process involves heating sheet metal to a predetermined temperature before quickly transfer to dies with an internal cooling system. This allows for simultaneous stamping and quenching of the sheet [7]. As hot stamping eliminates the need for die heating, it lowers the quality requirements for the dies are reduces heating and forming times. This significantly shortens the processing cycle, saves energy and protects the environment. Therefore, researching and exploring the hot stamping process for titanium alloys is highly beneficial [8]. Current research on the hot stamping of titanium alloys primarily focuses on Ti6Al4V alloy, which is the most widely used titanium alloy globally. To characterize its high-temperature flow behavior, extensive constitutive models have been developed in previous studies. Current research on the hot stamping of titanium alloys primarily focuses on Ti6Al4V alloy, the most used titanium alloy in the world today. To describe its high-temperature flow behavior, generous constitutive models have already been developed in previous studies. Gao et al. [9] constructed six constitutive models to describe the high-temperature flow behavior of Ti6Al4V alloy sheet under conditions ranging from 650 to 750 °C and discussed their accuracy. The results showed that the Hensel–Spittel (HS) model has the best prediction result while the Johnson–Cook (JC) model has the worst prediction result. Chao et al. [10] formulated the constitutive equations based on the stress–strain curves of Ti6Al4V alloy between 700 and 800 °C. The strain-compensated Arrhenius constitutive model showed high predictive capability under most experimental conditions. Yang et al. [11] developed a viscoplastic constitutive model for Ti6Al4V alloy, incorporating damage and softening mechanisms, and incorporating predicted the evolution of damage and fracture processes in hot tensile specimens. While constitutive models can predict the thermal flow behavior of materials, they are not directly applicable to guide hot forming process. In contrast, hot processing maps can characterize the processing performance of materials under various conditions, allowing for a more direct discussion of specific processing parameters. Qiu et al. [12] constructed hot processing maps for SP700 titanium alloy, verified microstructures, and identified optimal processing parameter for the alloy. Luo et al. [13] analyzed hot processing maps of Ti6Al4V alloy, noting significant variations in the instability parameter across different strain rates and deformation temperatures when the alloy was in α + β phase region. These studies demonstrate that constitutive models and hot processing maps are widely applied in isothermal hot forming of titanium alloys. However, few studies have specifically focused on the hot stamping process of titanium alloys under conditions with a wide temperature range.

Recent research has extensively explored the microstructural evolution during hot forming process of titanium alloys. Kopec et al. [14] studied the microstructural evolution of Ti6Al4V alloy during hot stamping, revealing phase transformations during heating and the formation of transformed β phase during forming. While the phase transition in hot stamping of titanium is well documented, the deformation mechanisms remain underexplored. Dynamic recrystallization (DRX) is a crucial method for enhancing the microstructure, plasticity, and processability of metallic materials during hot forming. Previous studies indicate that the softening behavior of titanium alloys during hot forming typically results from both DRX and dynamic recovery (DRV) [15,16]. Ning et al. [17] investigated the dynamic softening behavior of TC18 titanium alloy, finding that DRV and DRX dominate under different deformation conditions. Li et al. [18] found that during hot deformation of Ti-6Cr-5Mo-5V-4Al alloy in the α + β phase region, the DRX volume fraction is mainly influenced by strain and shows little sensitivity to deformation temperature and strain rate changes. The primary mechanisms of DRX include discontinuous dynamic recrystallization (DDRX) and continuous dynamic recrystallization (CDRX). An et al. [19] investigated the deformation mechanism of Ti-2.5Zr-2Al alloy in α + β phase region, finding that the CDRX mechanism-dominated DRX mechanism intensified with increased strain. As Ti6Al4V is the most widely used titanium alloy in the world today, the study of its hot mechanical properties has high application value. In addition, Ti6Al4V alloy, as a two-phase titanium alloy, has excellent mechanical properties, so the study of its hot stamping process is of great significance to the current lightweighting research. Despite extensive literature on softening mechanisms during hot deformation of titanium alloys, research on deformation mechanisms in Ti6Al4V alloy under hot stamping conditions remains incomplete, necessitating further study.

In order to achieve this objective, this paper investigated the flow behavior of Ti6Al4V alloy within temperatures of 500–800 °C and strain rates of 0.1 s^−1^–0.005 s^−1^. The strain-compensated Arrhenius constitutive model and a hot processing map are established. Based on hot processing map, the effects of processing parameters such as stamping speed, heating temperature and deformation amount on the quality in hot stamping of Ti6Al4V alloys were investigated, and the microstructural evolution of Ti6Al4V alloys under different deformation conditions was analyzed. And the relationship between the DRX mechanism and deformation amount in hot stamping forming of Ti6Al4V alloy was revealed, which provides theoretical support for precise control of microstructure and mechanical performance in Ti6Al4V alloy hot stamping during industrial production.

## 2. Materials and Experimental Details

### 2.1. Materials

**Figure 1:**
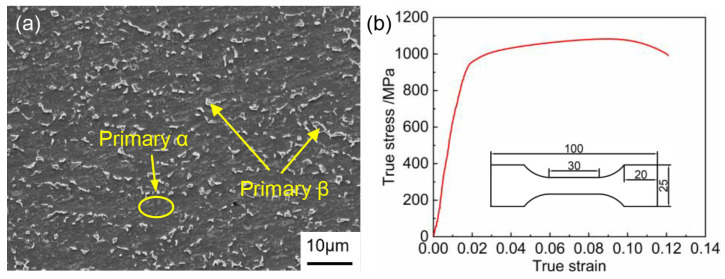
Initial microstructure (**a**) and room temperature tensile curve (**b**) of Ti6Al4V titanium alloy.

The material used in this paper is an industrial Ti6Al4V alloy (Baoji Titanium Industry Co., Ltd., Baoji, China) sheet with a thickness of 2 mm. Table 1 displays its chemical composition. Figure 1a illustrates the initial microstructure, consisting of equiaxed α grains with a small amount of β phase, constituting approximately 6.35% of the structure. Figure 1b shows the room temperature tensile curve of the sheet. The Ti6Al4V alloy sheet was found to have a tensile strength of 1083.19 MPa and an elongation of 10.23%.

### 2.2. Experimental Details

#### 2.2.1. The Hot Tensile Test

**Figure 2:**
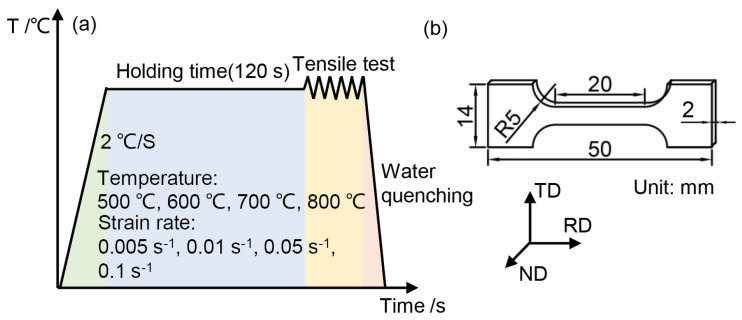
Schematic diagrams of the (**a**) hot tensile test process and (**b**) size of tensile specimen.

In order to explore the hot deformation behavior of a Ti6Al4V alloy sheet, the hot tensile test was conducted. The schematic diagram of the hot tensile test for sheet material is shown in Figure 2. Based on the existing research, in the hot stamping forming process of Ti6Al4V alloy, the deformation starting temperature is usually between 700 and 800 °C, and in current studies of hot stamping of Ti6Al4V alloy, the temperature range chosen is usually covered by 500–800 °C [14,20], so the deformation temperatures were set to be 500 °C, 600 °C, 700 °C and 800 °C, and according to the real hot stamping process, the strain rates were set to be 0.005 s^−1^, 0.01 s^−1^, 0.05 s^−1^ and 0.1 s^−1^. The specimens were subjected to 800 °C for 2 min air cooling heat treatment before the hot tensile test. In order to ensure even temperature division, the specimens were held for 2 min after heating to the target temperature.

#### 2.2.2. The Hot Stamping Test

The hot stamping experiment of Ti6Al4V alloy was divided into several stages. The first stage involved heating. To achieve a higher heating rate during deformation, this study utilized a heating furnace equipped with molybdenum–silicon rods, enabling rapid sheet heating to the target temperature. During heating, the sheet’s average heating rate was 14 °C/s, and the maximum rate was 60 °C/s. After heating to the target temperature, the sheet was held in the furnace for 2 min to ensure uniform temperature distribution. The second stage involved the forming stage. The sheet was then removed from the furnace and quickly transferred to room temperature, box-shaped part dies for stamping. The third stage involved holding pressure and quenching. After closing the dies, pressure was held for 1 min to reduce part springback. Before forming, the surface of the sheet was sprayed with boron nitride lubricant, and the top and bottom dies were coated with boron nitride grease to reduce friction.

**Figure 3:**
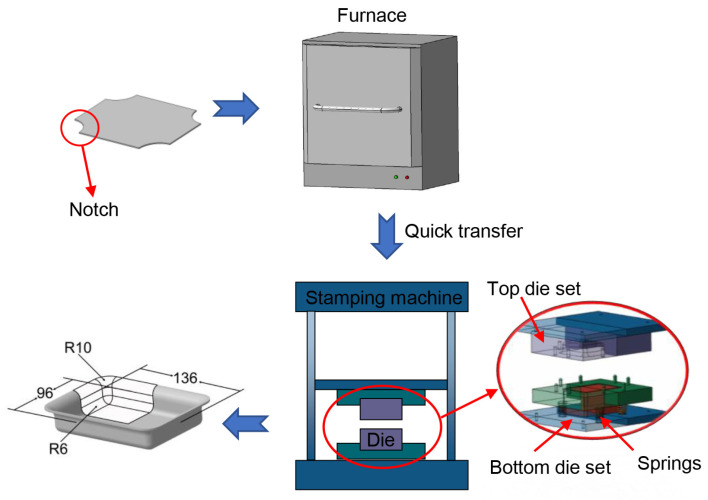
Demonstration of hot stamping formation process.

The whole forming process and the dies profile are shown in Figure 3. The sheet size was 210 × 170 × 2 mm. To reduce wrinkling, it is necessary to cut quarter-circle notches with a radius of 38 mm at four corners of the rectangular sheet. The sheet pressing force was set at approximately 10 kN. The formed part was a box-shaped component including the flange. To determine the optimal forming temperature, titanium alloy specimens with a size of 170 × 20 × 2 mm were previously subjected to hot stamping experiment. Holes were drilled in the sheet’s side, and thermocouples were inserted to measure the precise temperature changes during hot stamping experiment.

## 3. Results and Discussion

### 3.1. Thermal Rheological Behavior

**Figure 4:**
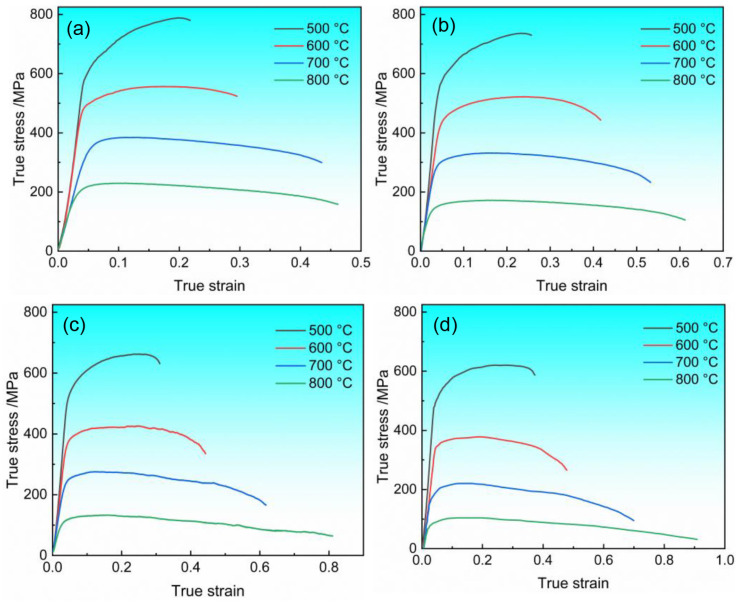
True stress–strain curves at strain rates of (**a**) 0.1 s^−1^, (**b**) 0.05 s^−1^, (**c**) 0.01 s^−1^, and (**d**) 0.005 s^−1^.

The true stress–strain curves of Ti6Al4V alloy sheet were obtained through hot tensile tests, as shown in Figure 4. As observed in Figure 4a–d, with increasing temperature and decreasing strain rate, the tensile strength of Ti6Al4V alloy decreases and the elongation increases, indicating enhanced material plasticity. During the initial loading stage, the flow stress rapidly increases, dislocation density significantly rises, and work hardening predominates. With increasing deformation, the continuous accumulation of dislocations leads to the activation of softening mechanisms such as DRV and DRX and their gradual enhancement [21,22]. Under different strain rates and temperature conditions, the materials display markedly different softening behaviors. The flow characteristics of hot deformation depend significantly on the competition between work hardening, DRV and DRX [23].

**Figure 5:**
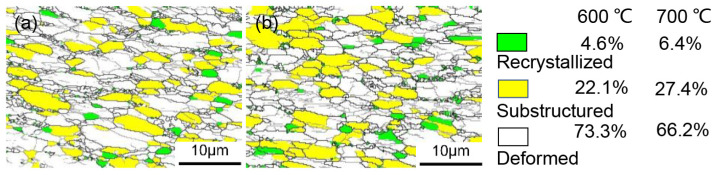
Crystal distribution map at temperatures of (**a**) 600 °C and (**b**) 700 °C.

Taking Figure 4a as an example, under the same strain rate, as the deformation temperature increases, the softening mechanisms are enhanced. When the heating temperature reached 600 °C, a balance between softening mechanisms and work hardening is achieved, leaving the flow stress unchanged with increasing strain. At a heating temperature of 700 °C, the softening mechanisms continue to strengthen, surpassing work hardening and resulting in material softening. To further investigate the causes of softening at elevated deformation temperatures, electron backscatter diffraction (EBSD) tests were conducted on the fractured sections of tensile specimens under a strain rate of 0.1 s^−1^ and heating temperatures between 600 and 700 °C in Figure 5. When the orientation difference between adjacent grain boundaries is less than 2°, grains are marked in white to indicate deformation. As hot processing proceeds, DRV action causes subgrain boundaries to gradually appear within deformed grains. Grains are marked in yellow when the orientation difference between adjacent grain boundaries ranges from 2 to 15°, indicating substructure grains. During deformation, low angle grain boundaries (LAGBs) continue to rotate, forming high angle grain boundaries (HAGBs). Grains are marked in green when the orientation difference between adjacent grain boundaries exceeds 15°, indicating recrystallized grains. At both 600 °C and 700 °C, a small number of fine DRX grains are produced, and the proportion of DRV grains significantly exceeds that of DRX, highlighting DRV’s dominant role in softening behavior [24]. However, at 700 °C, the percentage of both DRX and DRV grains is higher than at 600 °C, indicating that the material’s softening is due to the concurrent growth of DRX and DRV at elevated temperatures.

At a deformation temperature of 500 °C, significantly different softening behaviors were observed across various strain rates. The true stress–strain curves in Figure 4c,d show a brief equilibrium stage absent in Figure 4a,b. This occurs because lower strain rates extend deformation cycles, enabling DRX and DRV to significantly reduce dislocation density and enhanced dynamic softening effects. Conversely, higher strain rates result in greater dislocation densities, leading to enhanced work hardening effect [25]. Additionally, at lower temperatures, the resistance to dislocation motion increases, resulting in more pronounced work hardening [26]. This amplifies the enhancement of the softening effect as the strain rate decreases, leading to a significant difference in softening behavior under varying strain rates at lower temperatures.

### 3.2. Establishing the Constitutive Model

To describe the flow behavior of materials at high temperatures, Zener et al. [27] investigated the effects of temperature and strain rate on deformation to obtain Equation (1), and a further calculation able to produce Equation (2). In recent studies, the Arrhenius constitutive model has been widely used to describe the flow behavior of Ti6Al4V alloys at high temperatures. The expression can be expressed as:

(1) $$Z=\dot{\varepsilon }\exp(Q/RT)$$

(2) $$\ln Z=\ln A+n\ln\left[\sinh\left(\alpha \sigma \right)\right]$$

where *Z* is the Zener–Hollomon parameter, $\dot{\varepsilon }$ is the strain rate, *σ* is flow stress, *Q* is hot deformation activation energy, *R* is molar gas constant, *T* is the forming temperature, *A* is material constant, *α* is stress-level coefficient and *n* is stress exponent. Based on above parameters, the Arrhenius constitutive equation for Ti6Al4V alloy with strain of 0.06 can be expressed as:

(3) $$\dot{\varepsilon }=4.827\times 10^{10}{\left[\sinh\left(0.00203\sigma \right)\right]}^{12.0325}\exp\left[\left(-207859/RT\right)\right]$$

**Figure 6:**
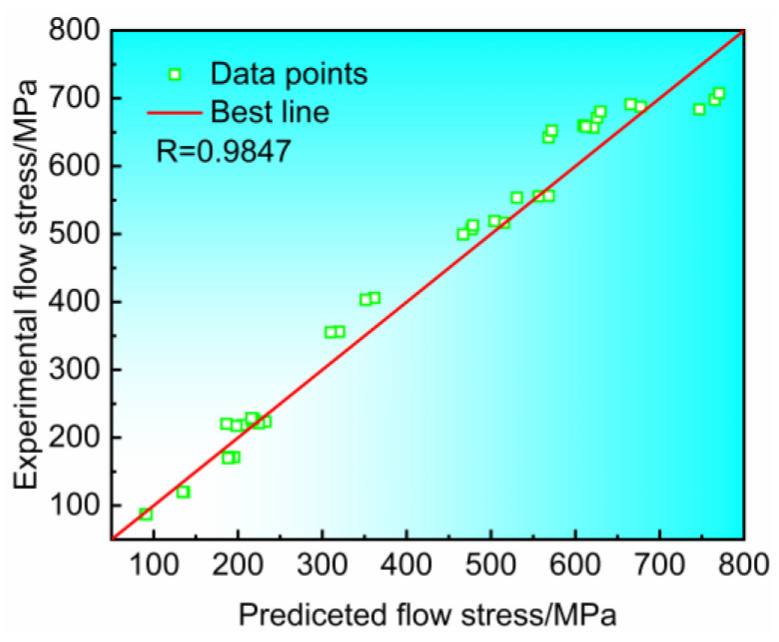
Ti6Al4V alloy predictor stress and experimental stress correlation.

However, the previous equation can not consider the influence of strain on the parameters. To improve the accuracy of the constitutive model, the values of *α*, *n*, *Q*, and ln*A* are calculated for strains of 0.06, 0.08, 0.1, 0.12, 0.04, 0.06, 0.08, and 0.2. The resulting strain-compensated Arrhenius constitutive model is shown in Table 2. The stress values were calculated according to the constitutive model and compared with the test values, as shown in Figure 6. The constitutive model produced an average error of only 8.3% in the stress–strain curve, and the correlation coefficient value *R* is as high as 0.9847 obtained after linear fitting, indicating that the constitutive model has a good prediction ability for the flow stress of Ti6Al4V alloy under a large temperature span.

### 3.3. Hot Processing Maps

Hot processing maps reveal the formability of materials under various deformation conditions and assist in optimizing hot processing parameters. The hot processing map consists of a power dissipation diagram based on the power dissipation efficiency factor η and the rheological instability diagram, based on the instability parameter ξ [28]. Rheological stresses with strain variables of 0.1 and 0.2 were selected to determine the power dissipation efficiency and instability coefficient, respectively. The strain rate sensitivity coefficient m is first calculated based on the stress level at specific temperature and strain rate conditions, and is defined by the following equation:

(4) $$m=\frac{\partial \ln\sigma }{\partial \ln\dot{\varepsilon }}=b+2c\ln\dot{\varepsilon }+3d{\left(\ln\dot{\varepsilon }\right)}^{2}$$

where *σ* is flow stress, $\dot{\varepsilon }$ is the strain rate, and *b*, *c* and *d* are material constants. The *m* value obtained from the solution is shown in Table 3. The value of *m* can determine the plasticity of the material. A larger *m* value indicates better plasticity of the material [29]. A negative of m suggests that the material is difficult to deform plastically. From Table 3, it can be observed that when strain is 0.1 and temperature is 500 °C, the *m* value is negative for all strain rate conditions, so the material is difficult to be formed under this deformation condition. A higher *m* value is obtained at higher temperatures and strain rates, indicating that the plasticity of the material improves with the increase in temperature and strain.

The power dissipation efficiency *η* is a parameter that describes the dissipative characteristics of the material, which can be expressed as:

(5) η=2m/(m+1)

The expression of instability parameter *ξ* is as follows:

(6) $$\xi (\dot{\varepsilon })=\frac{\partial \ln\left[m/(m+1)\right]}{\partial \ln\dot{\varepsilon }}+m$$

**Figure 7:**
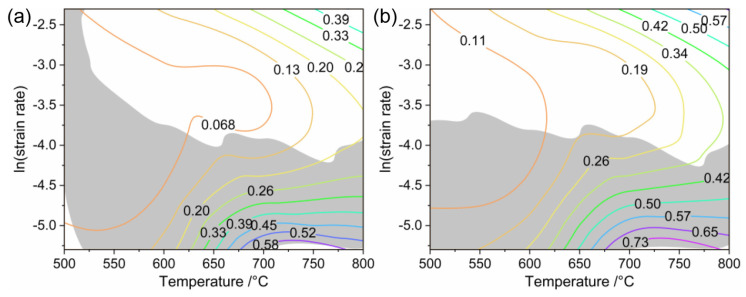
Processing maps of Ti6Al4V alloy at strains of (**a**) ε = 0.1 and (**b**) ε = 0.2.

When the instability coefficient *ξ* is negative, the material exhibits a higher tendency destabilization. Figure 7 shows the hot processing maps of Ti6Al4V alloy at different strain rates. In Figure 7, the gray area indicates the instability region, and the white area denotes the safe region. In general, at higher strain rates (>0.05 s^−1^), the material is less prone to instability, and the processing temperature range of the material is increased as the deformation amount increases. Within the temperature range of 500–550 °C, when the strain is 0.1, flow instability is likely to occur. When the strain increases to 0.2, the flow instability disappears in the low temperature region, enabling good processing performance even at lower temperatures.

### 3.4. Hot Stamping of Titanium Alloy

Based on the hot processing maps of Ti6Al4V alloy, a series of hot stamping experiments were conducted using custom-made box-shaped part dies to further investigate the influence of process parameters such as stamping speed, deformation temperature and deformation amount on the microstructure and forming quality of Ti6Al4V alloy.

#### 3.4.1. Effect of Stamping Speed on Hot Stamping

**Figure 8:**
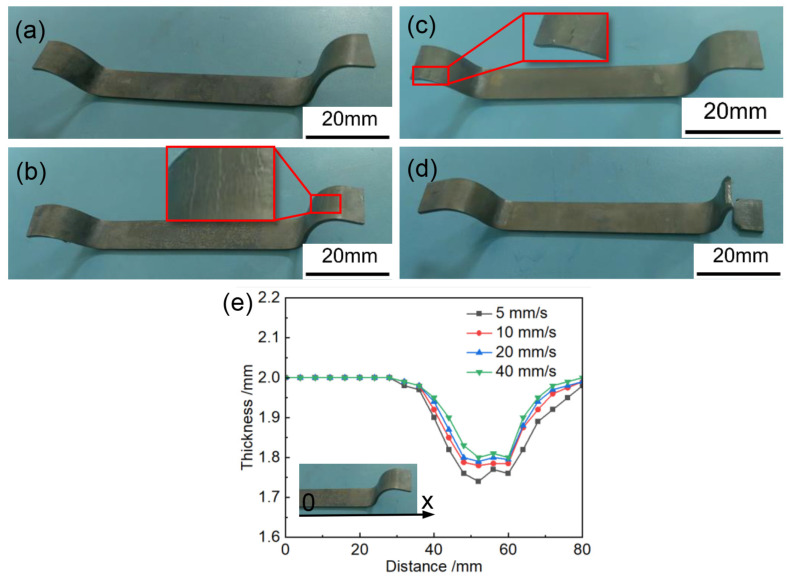
View of part formed at (**a**) 40 mm/s, (**b**) 20 mm/s, (**c**) 10 mm/s, and (**d**) 5 mm/s, and (**e**) variation in thickness.

**Figure 9:**
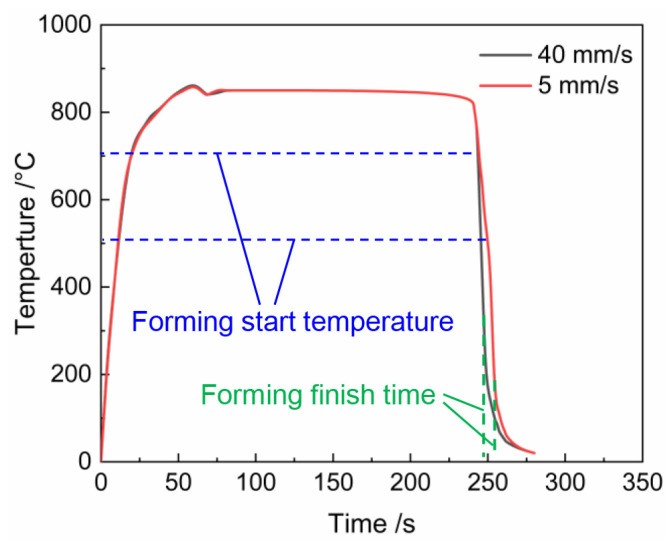
Evolution of temperature during forming process.

According to hot processing maps, it is observed that the Ti6Al4V alloy exhibits less instability tendency at higher deformation rates. To select an appropriate stamping speed, under the heating temperature of 850 °C [14], the stamping speed of press machine was adjusted to 40 mm/s, 20 mm/s, 10 mm/s, and 5 mm/s, respectively. The forming quality is shown in Figure 8. The temperature evolution of the sheet at stamping speeds of 40 mm/s and 5 mm/s is illustrated in Figure 9. At a stamping speed of 5 mm/s, the formed component fractures. Combined observations from Figure 9, the fracture is attributed to excessive temperature loss at a stamping speed of 5 mm/s, resulting in a forming temperature range of 200–500 °C, unsuitable for forming. At stamping speeds of 10 mm/s and 20 mm/s, while the components can be shaped, cracks appear on the rounded surface. At a stamping speed of 40 mm/s, the U-shaped part is formed perfectly. A study on part thickness shows that as stamping speed increases, the variation in sheet thickness decreases. This occurs because at higher stamping speeds, strain rate strengthening delays local deformation, leading to more uniform deformation of the sheet [20]. Therefore, it is difficult to obtain high-quality titanium alloy parts at lower stamping speeds, which is consistent with the hot processing maps provided in this paper. Therefore, in the hot stamping experiment of Ti6Al4V alloy, the stamping speed of the top die should not be lower than 40 mm/s.

#### 3.4.2. Effect of Temperature on Hot Stamping

Figure 9 shows that at a stamping speed of 40 mm/s, the temperature during the forming stage ranges from 693 to 384 °C. The hot processing maps indicate that instability is likely when the strain rate is between 0.1 s^−1^ and 0.05 s^−1^, and the deformation temperature is below approximately 525 °C. Therefore, to minimize the risk of instability at low temperatures, the heating temperature of the sheet was increased. Specimens were heated to 825 °C, 850 °C, 875 °C and 900 °C, respectively, before being stamped into components. It was found that components formed well of 825 °C, 850 °C, and 875 °C, with cracking occurring only at 900 °C.

**Figure 10:**
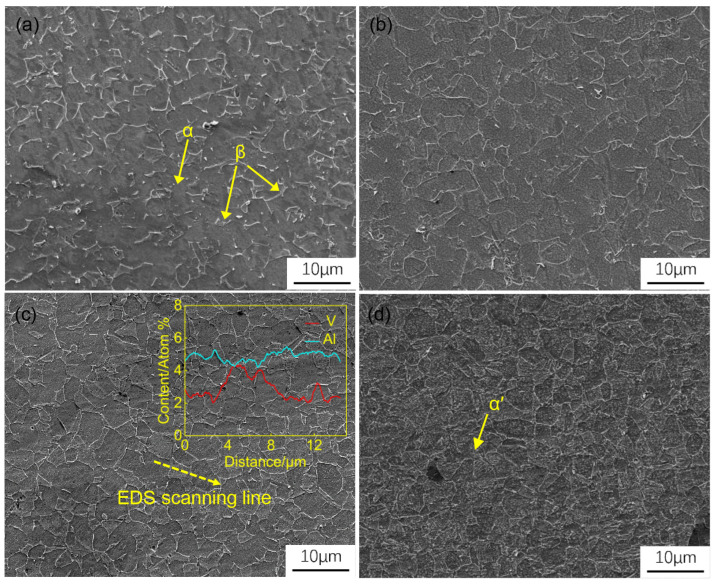
Microstructure images of part formed at (**a**) 825 °C, (**b**) 850 °C, (**c**) 875 °C, and (**d**) 900 °C.

The microstructure of the formed components is shown in Figure 10. As the heating temperature increases, the content of equiaxial α phase decreases, while β phase grains grow and their percentage increases. At heating temperatures of 825 °C and 850 °C, protuberances extending from the β phase grains can be observed. This occurs because the high cooling rate in cold die hot stamping forming leads to incomplete diffusion of alloying elements. V elements accumulate along α phase grain boundaries, causing morphological instability of some primary α phases at the phase interfaces [30]. At a heating temperature of 875 °C, the β phase grains grow and the protuberances extending are replaced by elongated β grains. The element distribution at 875 °C shows that the V element content is highest at the α-β grain boundaries, followed by within β grains. This indicates that with the increase in heating temperature, the atomic long-range mobility is enhanced, resulting in a higher diffusion capability. Consequently, a larger number of V atoms diffuse to α phase grain boundaries, expand β phase region in an enriched manner [31]. As shown in Figure 10d, it can be observed in the microstructure that there is already a small amount of α platelets has already precipitated in part of β matrix, which implies that the transformation of the β phase to the secondary α phase has already occurred in the hot stamping organization of Ti6Al4V alloy when the heating temperature reaches 900 °C [30]

**Figure 11:**
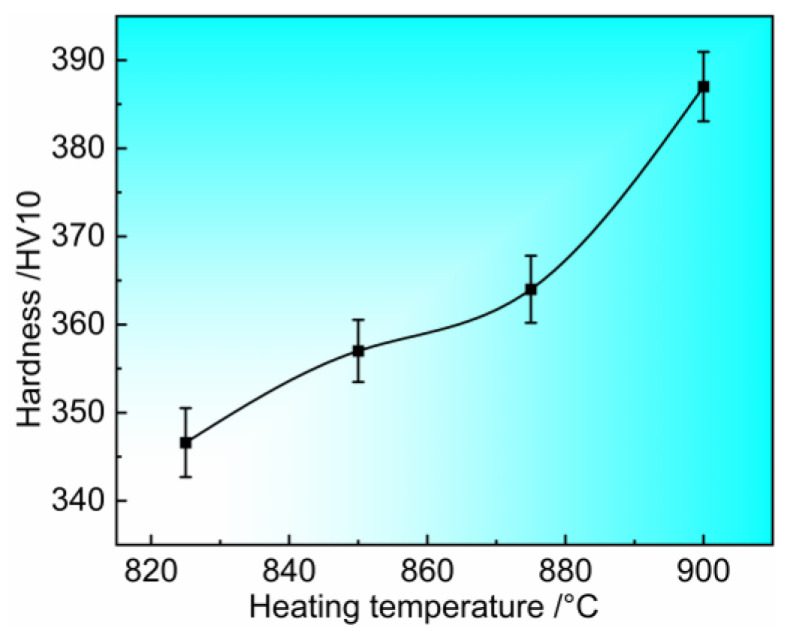
Post-form strength of components formed at different heating temperatures.

**Figure 12:**
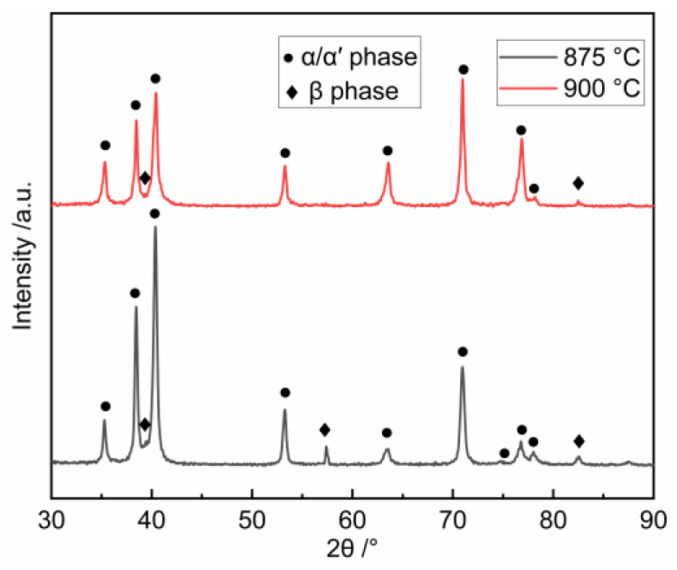
X-ray diffraction patterns for Ti6Al4V alloy under different heating temperatures.

The hardness of the formed parts was tested and the results are shown in Figure 11. It can be found that the hardness value of the formed parts increases from 347 HV at 825 °C to 387 HV at 900 °C. The hardness increases slowly when the heating temperature is between 825 °C and 875 °C, while the hardness increases significantly when the heating temperature is increased to 900 °C. Combined with the microstructure evolution in Figure 10, it indicates that phase transformation is the reason for the hardness enhancement. In order to further investigate the principle of hardness enhancement and probe the upper temperature limit of Ti6Al4V alloy hot stamping, X-ray diffraction patterns tests were carried out on the formed parts obtained under heating temperature of 875 °C and 900 °C, and the results are shown in Figure 12. The results show that only the diffraction peaks of α and β phases can be observed in Figure 12. Since the crystal structure of α′ phase and α phase are both hexagonal close-packed structure, the lattice constants of the two phases are very close to each other, so there is no difference in the position of the diffraction peak between α′ phase and α phase. The intensity of the diffraction peak of β phase at 875 °C is obviously higher than 900 °C, indicating that the transition from β phase to α′ phase is enhanced with the increase in the heating temperature.

**Figure 13:**
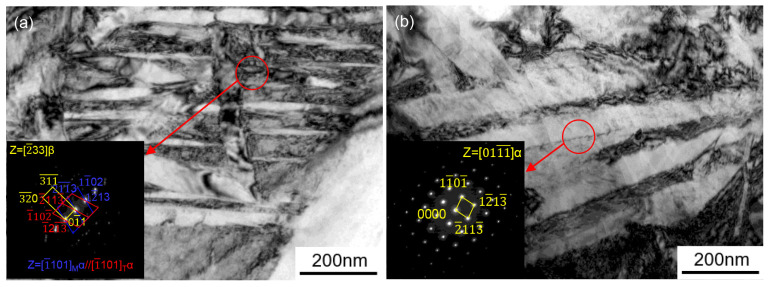
TEM micrograph of Ti6Al4V alloy after hot stamping at (**a**) 875 °C and (**b**) 900 °C.

In Figure 13, further transmission electron microscopy (TEM) tests of hot stamping organization at heating temperatures of 875 °C and 900 °C revealed that at a heating temperature of 875 °C, the β-transformed tissues obtained showed nano-sized martensite internally, with twinned α and β phases. However, when the heating temperature reaches 900 °C, the microstructure transforms into coarse slaty α′ phase, resulting in a significant increase in material hardness, which is the main cause of fracture. In Figure 13, the hardness increases more slowly within the heating temperature of 825–875 °C compared to 900 °C. Therefore, in order to achieve a higher initial forming temperature of the material, 875 °C is chosen as heating temperature for hot stamping.

#### 3.4.3. Effect of Deformation Amount on Hot Stamping

**Figure 14:**
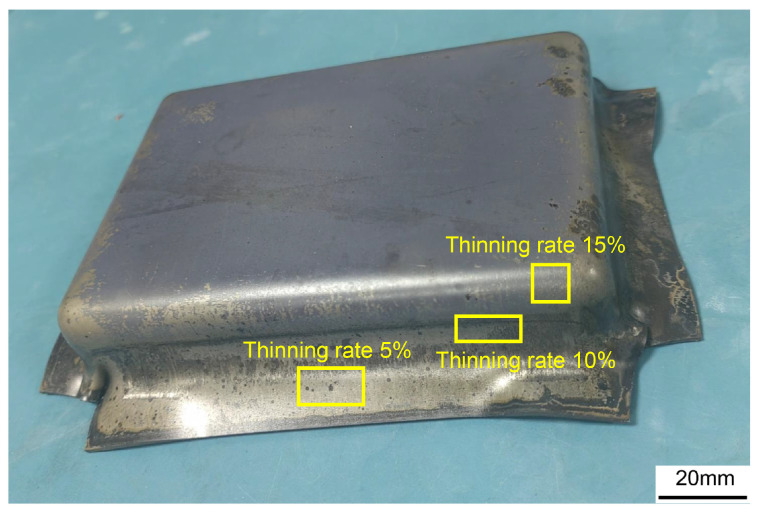
Forming parts and EBSD sample collection location.

Hot stamping experiments were conducted to explore the effects of deformation amount on microstructure. Figure 14 shows the box-shaped component formed by stamping under the conditions of a heating temperature of 875 °C and a speed of 40 mm/s. The formed box-shaped component shows no cracking and high forming quality.

Due to the difficulty in quantifying true strain experimentally, deformation amount is often measured using thinning rate. The thinning rate is the ratio of the difference in thickness before and after stamping to the original thickness of the sheet. Larger deformations correspond to higher thinning rates. Sampling was performed at positions with thinning rates of 5%, 10%, and 15% in the box-shaped components, as shown in Figure 14. The EBSD technique was employed to visually observe the distribution and morphology of substructures and characterize the microstructure of samples at different thinning rates.

**Figure 15:**
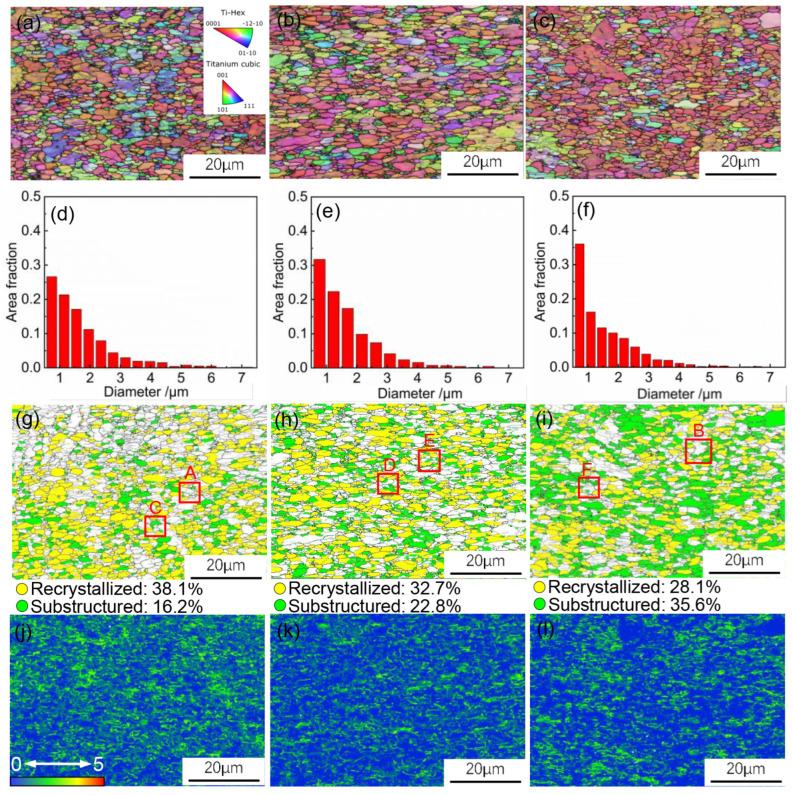
EBSD results of Ti6Al4V alloy for deflation of (**a**,**d**,**g**,**j**) 5%, (**b**,**e**,**h**,**k**) 10%, and (**c**,**f**,**i**,**l**) 15%.

Figure 15a–c depict inverse pole figure (IPF) of Ti6Al4V alloy at different thinning rates. A comparison between Figure 15a and c reveals that the thinning rate increases with the increase in the amount of deformation of the sheet. Figure 15d–f represent the grain size distribution at different thinning rates. The average grain sizes with thinning rates of 5%, 10%, and 15% were measured to be 1.73 μm, 1.68 μm and 1.57 μm, respectively. This indicates that during the hot stamping process of Ti6Al4V alloy, as the thinning rate increases, the average grain size decreases.

Figure 15g–i display the evolution of the microstructure with the level of thinning rate. As the thinning rate increases, the proportion of DRV grains remains relatively unchanged, and the proportion of DRX grains significantly increases. This indicates that DRX is the main cause of grain refinement. At thinning rate of 5% and 10%, the proportion of DRV grains is higher. This indicates that DRV is the primary softening mechanism at lower deformation amount. However, at a thinning rate of 15%, the proportion of DRX grains surpasses that of DRV grains. This suggests that at higher deformation amount, DRX surpasses DRV as the primary softening mechanism.

Figure 15j–l show the Kernel Average Misorientation (KAM) maps of Ti6Al4V alloy at different thinning rates. The KAM maps characterize the substructure density of the material, with higher values indicating greater defect concentration [32]. At a thinning rate of 5%, the substructure density is higher, indicating numerous defects like dislocations generated during deformation. As the thinning rate increases to 10% and 15%, the substructure density significantly decreases, indicating that many substructures generated by deformation are consumed This also confirms that the deformation mechanism in the hot stamping process is influenced by the amount of deformation. As deformation increases, the DRX mechanism is activated, leading to a decrease in dislocation density and grain refinement.

### 3.5. Analysis of the Dynamic Recrystallization Mechanism

**Figure 16:**
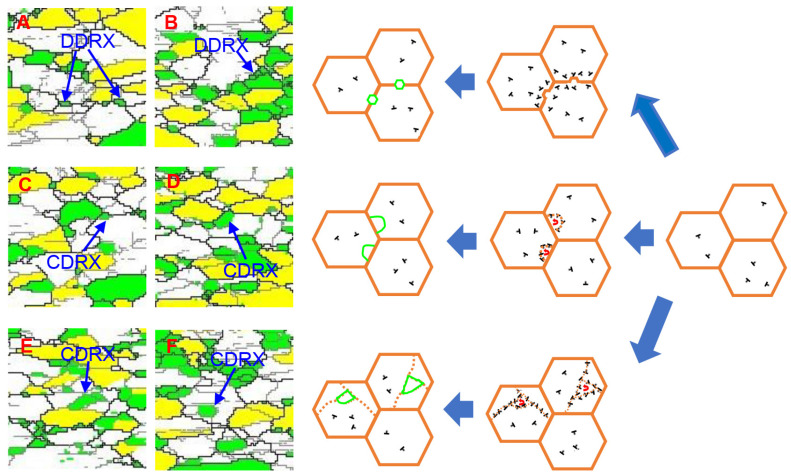
Schematic diagram of dynamic recrystallization formation: (**A**,**B**) DDRX grains and (**C**–**F**) CDRX grains.

In Ti6Al4V alloy, both DDRX and CDRX are common in deformation processes. Due to the significant impact of different DRX modes on practical production, it is necessary to analyze their mechanisms further. Figure 16 illustrates several typical DRX grain intercepted from Figure 15g–i. At area A and area B, DRX grains are generated at grain boundaries or triple intersections, which suggests that these DRX grains undergo nucleation and growth processes, a phenomenon that typically occurs in the DDRX mode [33,34]. In zones C and D, there are DRX grains within the original grains, bordering the original grains surrounded by fragments of LAGBs. This is due to the fact that DRX grains are formed by LAGBs and dislocation cells after continuous dislocation uptake without a nucleation process, known as CDRX [35]. At area E and area F, DRX grains are formed within the grains. This results from numerous dislocations into the interior under the influence of DRV, leading to dislocation cell formation within the original grains and subsequent transformation into CDRX grains [36]. The analysis above shows that both DDRX and CDRX exist in the microstructures across various deformation amounts.

**Figure 17:**
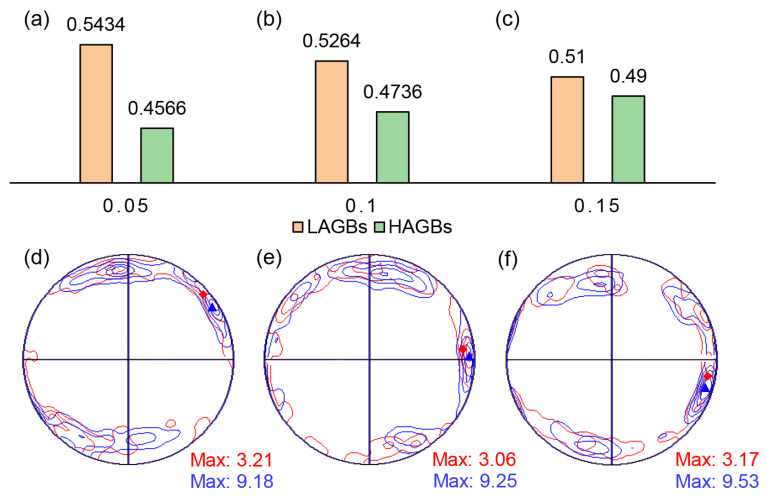
Evolution of recrystallized weaves at thinning rate of (**a**,**d**) 0.05, (**b**,**e**) 0.1, and (**c**,**f**) 0.15.

Figure 17a–c present the quantitative evolution in fractions of LAGBs and HAGBs with different thinning rates. It can be observed that as the thinning rate increases, the fraction of LAGBs decreases, while the fraction of HAGBs exhibits the opposite trend. This is due to the fact that lots of deformed substructures containing LAGBs are consumed during the formation of DDRX nucleation, resulting in a significant decrease in percentage of LAGBs [37]. It indicates that the DDRX mechanism is enhanced with the increase in thinning rate. Figure 17d–f illustrate the crystallographic orientation changes in DRX grains (mark in red lines) and remained grains (mark in blue lines) in {0001} pole figure at different thinning rates. There is little difference in texture between recrystallized and retained grains as the thinning rate increases. Given that DDRX has a distinct texture from the deformation texture, and CDRX’s texture is almost identical to the deformation texture [38], the similar between recrystallized and retained grain textures implies that CDRX consistently dominates this deformation mode. Additionally, DDRX processes often involve the growth of dynamically recrystallized grains, resulting in recrystallized grains of varying sizes and an uneven grain size distribution. Conversely, CDRX involves the continuous absorption of dislocations at subgrain interfaces, directly leading to the formation of recrystallized grains. Therefore, this process involves minimal growth of recrystallized grains, resulting in a more uniform and finer microstructure post-DRX completion. Statistical analysis of Figure 17d–f reveals that the average grain sizes of hot stamping components at thinning rates of 5%, 10%, and 15% are 1.73 μm, 1.68 μm, and 1.57 μm, respectively. The percentage of grains with diameters smaller than 1 μm are 26.62%, 31.74%, and 36.05%, respectively. This indicates that deformation increases, so does the proportion of fine grains, further supporting the dominance of CDRX during deformation, which intensifies with increased deformation.

In summary, both CDRX and DDRX mechanisms coexist in hot stamping of Ti6Al4V alloys. With increased deformation in the hot stamping process, the DDRX mechanism is enhanced to some extent, but the CDRX mechanism consistently dominates. Therefore, designing a rational deformation path and mode for the hot stamping of Ti6Al4V alloy components is crucial to achieving a finer and more uniform microstructure, which leads to enhanced mechanical properties.

## 4. Conclusions

Based on the stress–strain curve, a constitutive model for strain compensation and a hot processing map for Ti6Al4V alloy under a large temperature span in the hot stamping process were constructed. The hot stamping rheological behavior and the deformation mechanism of Ti6Al4V alloy were systematically studied. The main conclusions are as follows:

1. The stress–strain curve indicated that with the increase in temperature and the decrease in the strain rate, the rheological softening effect was enhanced, and the material exhibited temperature and strain rate sensitivity. A strain-compensated Arrhenius constitutive model suitable for hot stamping of Ti6Al4V alloy was established, which showed good agreement with experimental values. The average error of the stress–strain curve calculated using the constitutive model was only 8.3%.
2. A hot processing map suitable for the hot stamping process of Ti6Al4V alloy was established. The instability region mainly concentrates in the low strain rate region. Ti6Al4V alloy was less prone to instability when the strain rate was higher than 0.05 s^−1^, and the processing temperature range of the material was increased as the deformation amount increases. Based on the hot processing map, in hot stamping experiments, Ti6Al4V alloy parts can be successfully formed at stamping speeds greater than 40 mm/s and heating temperatures in the range of 825–875 °C.
3. The study of recrystallization mechanisms in Ti6Al4V alloy during hot stamping revealed that increased deformation has an exciting effect on DRX. When the thinning rate of Ti6Al4V alloy during hot stamping was increased from 5% to 15%, DRX was significantly enhanced, dislocation density was reduced, and grain refinement occurred. The characteristics of substructure and texture evolution indicated that both CDRX and DDRX coexist throughout the hot stamping of titanium alloy. With increasing deformation during hot stamping, the DDRX mechanism was strengthened, but the CDRX mechanism remained dominant.

## Figures and Tables

**Table 1:** Chemical composition of Ti6Al4V alloy (%).

Element	Ti	Al	V	Fe	Si
wt%	Bal	6.85	4.00	0.25	0.22

**Table 2:** Polynomial fitting results of α, *n*, In*A* and *Q*.

	*α*	*n*	*Q*	ln*A*
k_0_	−0.0103	15.96865	676.94558	87.48507
k_1_	0.76216	−507.81285	−22,345.4023	−3034.32718
k_2_	−18.23749	9887.6117	419,169.9129	58,534.95292
k_3_	222.92064	−101,148.339	−4,010,947.11	−582,395.4021
k_4_	−1474.3765	560,983.548	20,473,000	3,120,280.7897
k_5_	5015.36434	−159,233	−52,513,400	−8,506,609.959
k_6_	−6865.6785	1,809,399	52,517,400	9,217,898.9467

**Table 3:** Values of m calculated at different hot tensile condition.

ε	0.1				0.2			
T/°C	500 °C	600 °C	700 °C	800 °C	500 °C	600 °C	700 °C	800 °C
0.1 s^−1^	−0.19	0.21	0.39	0.61	0.057	0.14	0.29	0.49
0.05 s^−1^	−0.21	0.078	0.13	0.31	0.033	0.066	0.10	0.28
0.01 s^−1^	−0.20	0.052	0.26	0.30	0.049	0.078	0.28	0.318
0.005 s^−1^	−0.17	0.17	0.63	0.60	0.088	0.16	0.61	0.56

## Data Availability

Datasets generated and/or analyzed during the current study are available from the corresponding author on request.
